# Surgical Treatment Options for the Young and Active Middle-Aged Patient with Glenohumeral Arthritis

**DOI:** 10.1155/2012/846843

**Published:** 2012-03-22

**Authors:** Sanjeev Bhatia, Andrew Hsu, Emery C. Lin, Peter Chalmers, Michael Ellman, Brian J. Cole, Nikhil N. Verma

**Affiliations:** Division of Sports Medicine, Department of Orthopaedic Surgery, Rush University Medical Center, 1611 W. Harrison Street, Suite No. 300, Chicago, IL 60612, USA

## Abstract

The diagnosis and treatment of symptomatic chondral lesions in young and active middle-aged patients continues to be a challenging issue. Surgeons must differentiate between incidental chondral lesions from symptomatic pathology that is responsible for the patient's pain. A thorough history, physical examination, and imaging work up is necessary and often results in a diagnosis of exclusion that is verified on arthroscopy. Treatment of symptomatic glenohumeral chondral lesions depends on several factors including the patient's age, occupation, comorbidities, activity level, degree of injury and concomitant shoulder pathology. Furthermore, the size, depth, and location of symptomatic cartilaginous injury should be carefully considered. Patients with lower functional demands may experience success with nonoperative measures such as injection or anti-inflammatory pharmacotherapy. When conservative management fails, surgical options are broadly classified into palliative, reparative, restorative, and reconstructive techniques. Patients with lower functional demands and smaller lesions are best suited for simpler, lower morbidity palliative procedures such as debridement (chondroplasty) and cartilage reparative techniques (microfracture). Those with higher functional demands and large glenohumeral defects will usually benefit more from restorative techniques including autograft or allograft osteochondral transfers and autologous chondrocyte implantation (ACI). Reconstructive surgical options are best suited for patients with bipolar lesions.

## 1. Introduction

While the cause of primary glenohumeral osteoarthritis is largely unknown, secondary osteoarthritis is often due to trauma, acute or recurrent dislocation, or prior surgery. Primary glenohumeral arthritis typically results in posterior glenoid wear with posterior humeral head subluxation occurring in up to 50% of affected shoulders. Rotator cuff tears occur in less than 5–10% of cases of primary osteoarthritis. Joint space narrowing occurs with periarticular osteophyte formation most commonly on the inferior aspect of the humeral head. As a result, the anterior soft tissues such as the capsule and subscapularis become contracted and stiff, limiting external rotation [1]. With the growing elderly population in the US, the number of total shoulder arthroplasties performed each year has doubled over the past decade to approximately 20,000 cases [2]. While glenohumeral osteoarthritis typically affects older patients, in some cases it can affect younger, active patients causing significant pain and disability [3, 4].

The role of glenohumeral chondral lesions in the natural history of shoulder arthritis has not been well established, as most cartilaginous lesions of the glenohumeral joint are found incidentally and are well tolerated in young individuals. The diagnosis and treatment of symptomatic chondral shoulder lesions in young and active middle-aged patients is challenging and lacks a clear diagnostic algorithm. Often times the diagnosis is only reached when all other shoulder pathologies and causes of glenohumeral pain have been considered [5]. There are multiple sources of shoulder pain which must be considered in addition to cartilage lesions including labral pathology, biceps tenosynovitis, rotator cuff pathology, infection, and loose bodies.

Chondral lesions of the glenohumeral joint are not uncommon and have been found incidentally in 4.5–8.5% up to 17% of middle-aged patients with full-thickness rotator cuff tears during diagnostic arthroscopy [6–8]. Cartilage injuries may occur on the humeral head, glenoid, or both. These lesions often result after traumatic injury, arthroscopic surgery, osteochondritis dissecans, infection, chondrolysis, or avascular necrosis [9]. Most commonly, however, lesions may result as an early manifestation of degenerative arthritis. Chondral lesions have been noted in previous reports ranging from minor cartilage lesions such as fraying or thinning to complete cartilage loss [7]. One of the main difficulties in managing these chondral lesions is determining if they are a pain generator as even large lesions can be well tolerated. In addition, treatment is challenging as shoulder arthroplasty is not ideal in a younger age group despite its pain relief and restoration of function.

Initial treatment for symptomatic chondral lesions consists of nonoperative management with activity modification, steroid injections, and physical therapy [10]. If these therapies fail, there are a limited number of surgical options available. Previous studies have investigated the long-term outcomes of total shoulder arthroplasty and noted good clinical success, particularly older patient with glenohumeral degenerative disease and an intact rotator cuff—in one series by Torchia et al., pain relief was achieved in 83% and implant survival was 93% after 10 years [11]. In young and middle-aged patients, clinical success has been mixed. Sperling et al., in a recent series of 33 patients with a mean age of 46 years at the time of TSA, reported a 38% incidence of glenoid component failure requiring revision surgery [12]. Non-arthroplasty treatment strategies for glenohumeral arthritis have been devised in effort to postpone the need for total shoulder arthroplasty and avoid the incidence of early glenoid loosening—these techniques are integral to the treatment of younger patients with painful chondral lesions.

In place of arthroplasty, arthroscopic treatments have been used including debridement, chondroplasty, capsular release, biceps tenotomy or tenodesis, and subacromial decompression, and more recently reparative techniques including microfracture, autologous chondrocyte implantation, or osteochondral grafting [13]. However, the outcomes of these treatments are highly variable in regards to pain relief and restoration of function. The purpose of this paper is to examine the evaluation and management of glenohumeral chondral lesions and discuss the surgical treatment options for young and middle-aged patients.

## 2. Methods

A comprehensive Pubmed search was performed using the following Boolean search terms: glenohumeral arthritis, glenohumeral osteoarthritis, management, young patients. The yield of over 80 articles was examined carefully with emphasis placed on publications focusing on non-arthroplasty alternatives. These articles served as the basis for this paper in addition to our clinical experience.

## 3. Patient Evaluation

As noted by Gartsman et al., patients with glenohumeral arthritis will generally reveal complaints of pain, disability, and loss of quality of life which may be common to many types of shoulder pathology [4]. Patient symptoms are often of nonspecific shoulder pain and mechanical complaints [5]. The examiner should elicit a history of glenohumeral trauma or instability, activities, and arm positions that cause pain, effusions, neurological complaints, and prior operative and nonoperative shoulder interventions [14]. The examiner should have a general understanding of the demand a patient places upon their shoulder, including their athletic activities/aspirations and occupational demands. A history of instability may be particularly valuable given the connection between prior subluxation and dislocation events and the subsequent degeneration of the glenohumeral joint [15]. Generally the diagnosis of a chondral injury is one of exclusion given its relative rarity in comparison to lesions of the biceps tendon, subacromial space, and capsulo-labroligamentous complex in younger patient cohorts [5, 9]. If prior surgery has been performed, a review of operative notes and intraoperative pictures may be helpful in determining the prior procedures performed and the status of the articular cartilage at the time of previous arthroscopy.

Examination should include a general shoulder evaluation, with documentation of appearance, tenderness to palpation, range of motion, strength, and neurovascular status. Capsular contracture with loss of range of motion is common in cartilage injuries. Pain at full abduction and flexion is suggestive of impingement, while pain with the shoulder in mid-abduction or mid-flexion is more suggestive of a chondral lesion [5, 9]. Physical exam of the young patient with a glenohumeral cartilage injury can be complex due to the variety of possible pain-generating structures within the degenerative shoulder joint, including the glenoid labrum, the long head of the biceps tendon (an intra-articular but extrasynovial structure), the rotator cuff, and the glenoid or humeral articular cartilage [13]. In a degenerated joint, provocative maneuvers attempting to query any of the structures may be positive. As a result, glenohumeral chondral lesions can be easily confused with pain secondary to primary pathologies of these structures. In particular, patients with glenohumeral arthritis may have similar physical exam findings to those with subacromial impingement [8]. In an effort to differentiate patients with positive impingement signs between those with subacromial impingement and those with glenohumeral arthritis, Ellman and colleagues (1992) described the “compression-rotation” test in which the abducted humerus is axially loaded while gently internally and externally rotating. This test should cause pain in patients with glenohumeral arthritis but not those with subacromial impingement [4, 8]. Injection of the glenohumeral or subacromial space with congruent elimination of symptoms due to pathology in these respective spaces may be particularly helpful.

Radiographic evaluation of the patient with shoulder pain should include anteroposterior and lateral views in the scapular plane with axillary views. Several views have been developed for specific evaluation of the glenoid, which may be progressively deformed in glenohumeral arthritis, including the West Point, apical oblique, and Didiee views. The Stryker notch view may be used to evaluate for a Hill-Sachs lesion. While these views are relatively nonspecific for chondral injuries, they can be used to grade glenohumeral arthritis using a system developed by Samilson and Prieto based upon the size of the glenoid or humeral osteophytes [16]. Computed tomography, especially with 3D reconstruction and digital subtraction of the humeral head, offers a more complete evaluation of the osseous anatomy of the glenohumeral joint and presence, if any, of glenoid bone loss [17]. Magnetic resonance imaging (MRI) can best characterize the status of the soft tissues, including the rotator cuff and glenohumeral ligaments. Relative to the knee, a shoulder MRI is less sensitive for chondral lesions because of the thin cartilaginous covering in the shoulder [18]. Because these imaging studies remain imperfect for the diagnosis of chondral injuries in the glenohumeral joint, diagnostic arthroscopy remains the gold standard and may be appropriate to offer in cases of diagnostic uncertainty despite comprehensive noninvasive evaluation and failure of conservative care [5, 9].

## 4. Surgical Decision Making

When treating patients with glenohumeral chondral pathology, identification of patients with *symptomatic* cartilaginous lesions and appropriate surgical decision making is paramount for success [5]. Cartilage defects are frequently encountered on imaging or arthroscopic exam. At the outset, it is essential to identify those patients whose pain is generated by these lesions and differentiate incidental chondral lesions from symptomatic ones. Shoulder impingement, commonly seen in younger age groups, frequently mimics symptomatic glenohumeral chondral pathology but is frequently differentiated from chondral lesions with the help of the compression-rotation test [8]. A history of shoulder trauma, previous shoulder surgery, recurrent subluxations or dislocation, mechanical symptoms (catching or clicking), and persistent pain after subacromial diagnostic injection are other clues that suggest symptomatic chondral pathology [19]. In our experience, symptomatic chondral lesion should be considered a diagnosis of exclusion, with all other sources of shoulder pathology treated first, and most reparative procedures should be considered as a secondary option.

Once glenohumeral cartilage pathology is felt to be the source of pain, a methodical approach should be used for treatment. Correct surgical or nonsurgical management depends on the patient's functional demands, co-morbidities, symptoms, age, occupation, degree of injury, and concomitant shoulder pathology [5]. Furthermore, the size, depth, and location of symptomatic cartilaginous injury should be carefully considered. Most patients will have cartilage pathology limited to the glenoid or humeral head (a unipolar lesion), but some may have pathologic lesions on both chondral surfaces (bipolar lesions). The presence of bipolar chondral pathology should be prudently noted as surgical treatment options are different from that for unipolar lesions.

Older patients who are poor surgical candidates yet have symptomatic glenohumeral chondral lesions are well suited for nonoperative modalities which relieve pain and maintain shoulder function. Conservative options for treatment include physical therapy, judicious intra-articular steroid injections, and topical or oral nonsteroidal anti-inflammatory drugs. Glenohumeral viscosupplementation has sometimes been used in an off-label fashion and may have a role in providing relief [19–21]. If conservative options fail in an older age patient with limited shoulder demands, total shoulder arthroplasty offers the most reproducible long term outcome with regard to functional improvement and pain relief. On the other hand, arthroscopic management may be more fitting for younger, more active patients who are willing to comply with postoperative rehabilitation and desire to maintain high shoulder demand activities [19]. In these instances, surgical management is broadly classified into palliative, reparative, restorative, or reconstructive techniques [5].

**Figure 1:**
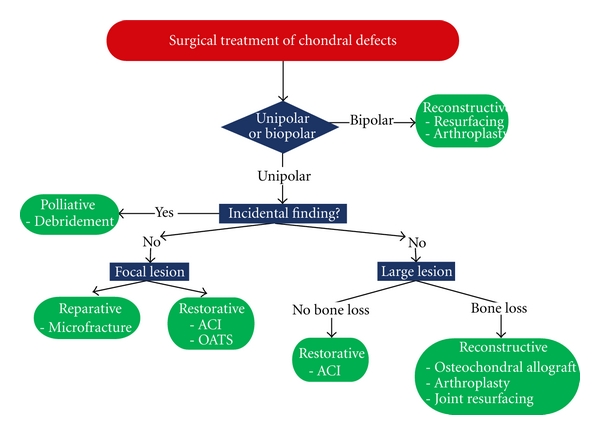
Approach to surgical decision making in patients with chondral defects of the glenohumeral joint. Adapted from [5].

 A general approach to surgical management of patients with symptomatic glenohumeral chondral lesions is outlined in Figure 1. Patients with lower functional demands and smaller lesions generally tend to have more success with simpler, low morbidity palliative procedures such as debridement (chondroplasty) and cartilage reparative techniques (microfracture). Those with higher functional demands and large glenohumeral cartilage defects will usually benefit more from cartilage restorative techniques including autograft or allograft osteochondral transfers and autologous chondrocyte implantation (ACI). Reconstructive surgical options are best suited for patients with bipolar lesions.

## 5. Palliative Treatment Strategies

The mainstay of palliative surgical treatment is arthroscopic debridement and lavage (chondroplasty). Debridement acts to alleviate irritating mechanical symptoms that arise from edge instability. Additionally, arthroscopic debridement stabilizes cartilage lesions, thereby reducing the risk of further delamination.

 The ideal patient for chondroplasty is a low demand, elderly individual (>65 years of age) who presents with a small chondral defect, typically less than 2 cm^2^ in size [22]. In performing chondroplasty, a motorized shaver should be used to gently debride the cartilaginous lesion down to a stable, vertically oriented rim of cartilage with subchondral bone at its base. Every attempt should be made to sculpt vertical chondral walls—as shown by Rudd and colleagues, chondral lesions with vertically oriented walls had significantly less defect progression than lesions with walls beveled at 45 degrees [23]. In addition to debridement, concomitant shoulder pathologies such as impingement, AC joint arthrosis, rotator cuff tears, and biceps or labral pathology should be addressed simultaneously. In settings of diffuse osteoarthritis, chondroplasty may improve shoulder pain by reducing joint irritation and synovitis and stabilizing the residual articular surface from further cartilage delamination [19]. In addition, most patients demonstrate restricted range of motion associated with progressive cartilage degeneration and may benefit from capsular release and resection of osteophytes.

 Reported results of chondroplasty suggest that the procedure is beneficial in a majority of patients indicated for surgery. In a series of 71 patients undergoing arthroscopic debridement of glenohumeral chondral lesions, Van Thiel et al. noted significant improvement with regards to the American Shoulder and Elbow Surgeon (ASES) score, simple shoulder test, and visual analog pain score after surgery [13]. The authors ultimately concluded that arthroscopic debridement of glenohumeral degenerative joint disease is effective but has an increased risk of failure in the presence of grade IV bipolar disease, joint space less than 2 mm, and large osteophytes [13]. Similarly, Cameron et al. found significant improvement with regard to pain and function in 54 out of 61 patients with small, full-thickness chondral defects [22].

## 6. Reparative Treatment Strategies

For well-contained small unipolar cartilage lesions, marrow stimulation techniques are the next level of treatment that may be indicated beyond debridement. Microfracture, a cartilage reparative strategy popularized by Steadman et al. [24] in the knee, is a first-line technique for stimulating fibrocartilage growth in a chondral defect as a means of providing structural support to surrounding tissue [5, 9, 24]. The procedure can be performed arthroscopically in the humerus or glenoid and does not restrict opportunities for cartilage restoration in the future. However, differences in articular cartilage between the shoulder and knee are significant, which may impact results following microfracture. The thickness of the articular cartilage in the shoulder is much thinner than the knee, with maximum thickness of 1.5 mm tapering to less than 1 mm at the periphery of the humeral head and center of the glenoid [11]. In addition, the convex shape of the humeral head and often peripheral location of articular defects on the glenoid may limit the ability to contain the initial clot.

 Although the procedure can be performed in a simple manner, various technical pearls are critical for success. After identifying the relative boundaries of the chondral defect using a motorized shaver or curette, the lesion should be thoroughly debrided to produce vertically oriented walls. By doing so, the lesion is much better contained and added stability is provided to the neighboring chondral tissue. Next, the calcified cartilage layer should be thoroughly removed off of the subchondral bone using a curette. A sharp awl is then used to penetrate subchondral bone multiple times as perpendicularly to the surface as possible—each hole should be approximately 2-3 mm apart and should produce bleeding when the pump is turned off [19]. It is imperative that a visual confirmation of subchondral bleeding is noted—bleeding elements contain various mesenchymal stem cells, growth factors, and growth proteins that come together to generate fibrocartilage. Once the procedure is over, a protected loading and motion protocol should be enacted to promote a healing response [5].

 Although outcomes of microfracture techniques have mostly been reported in knee literature, various authors have described their effectiveness in the shoulder (Table 1). In a case series of 16 patients undergoing microfracture of glenoid or humeral head chondral defects, Frank et al. noted significant improvement in ASES score, SST score, and visual analog pain score at a mean followup of 27.8 months [18]. Three of the sixteen patients, however, did ultimately fail the treatment and went on to further shoulder surgery. Millet and colleagues examined 25 patients undergoing arthroscopic microfracture and also found statistically significant improvements in pain, ASES score, ability to work, activities of daily living, and sporting activities following surgery [25]. Interestingly, the authors noted that their best results occurred in patients with unipolar lesions of the humeral head [25].

## 7. Restorative Treatment Strategies

The overall purpose of restorative surgery for glenohumeral chondral lesions is to anatomically reestablish damaged or missing cartilage. The primary surgical techniques are osteochondral grafting using autograft or allograft and autologous chondrocyte implantation (ACI). Both of these treatments necessitate open surgery and have potential complications such as donor site morbidity and the need for multiple surgeries (ACI) [5]. Therefore, patient selection is critical to overall outcomes and patient satisfaction.

The ideal patient for osteochondral grafting or ACI is young to middle aged, physically active on a regular basis, with an isolated humeral focal chondral defect. Use of osteochondral autograft has been previously well described for chondral lesions in the knee but to a much lesser extent in the shoulder. Scheibel et al. examined a series of eight grade IV humeral head chondral lesions (average size 150 mm^2^) in which he performed osteochondral grafting from the knee [28]. The authors reported significant clinical improvement in pain and function at 33 months followup. Postoperative shoulder MRIs showed graft incorporation in all but one patient, and donor site morbidity from the knee was noted in two patients. An explanation for donor site morbidity is that knee contact pressures at osteochondral donor sites are increased, particularly when taken from the lateral condyle and central trochlea, which limits the amount of cartilage available [29]. For deep, extensive, and uncontained glenohumeral chondral lesions, osteochondral grafting using size-matched allograft from the humeral head or glenoid remains the best option [5].

Proper size matching is critical when performing osteochondral grafting, and plugs can vary in size, up to 40 mm according to some reports [30]. As outlined by Cole et al., there are several general principles that should be followed when performing osteochondral allografting [5]. The first is appropriate handling and use of fresh cartilage graft tissue. There is optimal cell viability when fresh grafts are used within 28 and, ideally, within 21 days of harvest as allografts have shown a predictable decline in cell viability, cell density, and metabolic activity with time [31, 32].

Humeral or glenoid size matching is performed using plain radiographs of the shoulder and size markers to correct for machine-dependent magnification. In addition, a thin shell allograft plug that preserves cartilage integrity significantly decreases the amount of subchondral bone used and the antigenicity of the graft [33]. Potential immunogenic responses can further be reduced during the procedure by using pulsatile lavage of the graft prior to implantation. Lastly, while press-fit allograft implantation can be achieved, fixation can be strengthened using bioabsorbable screws or pins.

Outcome studies of full-thickness cartilage defects of the knee treated by ACI show good to excellent results at long-term followup [34]. In regards to the glenohumeral joint, ACI remains controversial and unproven. There has been a published case report of a teenage athlete treated with ACI of the humeral head using a tibial periosteal graft and a two-stage technique with knee cell growth and shoulder implantation [35]. At one year followup, the patient had no pain and full range of motion without functional loss.

## 8. Reconstructive Treatment Options

As opposed to restorative treatment options for smaller, superficial chondral defects, reconstructive cartilage surgery may be required for large and deep unipolar or bipolar defects. Reconstructive surgery is considered a “salvage” treatment option, with goals of restoring durable function to the shoulder and decreasing pain, typically as a final attempt before considering total shoulder arthroplasty (TSA) or glenohumeral arthrodesis. While reconstructive surgery technically includes hemiarthroplasty or TSA, we will focus on the prearthroplasty management for young and middle aged patients with significant chondral defects. Prearthroplasty reconstructive surgery includes resurfacing techniques to the humeral head and glenoid, as well as biologic or nonbiologic interpositional arthroplasty techniques using a variety of tissues, such as meniscal allografts, anterior capsule, fascia lata, Achillestendon allograft, and other specialized matrices. Suggested potential benefits of biologic glenoid resurfacing include pain relief and improved range of motion similar to TSA, without the well-known complications of polyethylene wear, cement fragmentation, and glenoid loosening or dissociation.

### 8.1. Resurfacing Arthroplasty

For young and middle-aged active patients, there is a desire to avoid hemiarthroplasty or TSA due to high failure rates associated with progressive glenoid erosion or glenoid component loosening (~40%) [12]. Therefore, over the past couple years, attempts to compromise between bone-preserving procedures and arthroplasty have led to the development of new stemless implants, particularly in the humerus, which have become promising options if palliative procedures fail [19]. Similar to resurfacing procedures in the hip, however, the use of resurfacing arthroplasty/hemiarthroplasty in the shoulder is controversial. Stemless implants preserve anatomy and leave open various options for subsequent revision surgery in the future, but short- and long-term outcome studies are currently lacking. In addition, the use of a resurfacing device complicates the surgical approach to the glenoid limiting options for glenoid resurfacing or replacement and may result in high rates of persistent pain or progressive glenoid erosion similar to tradition hemiarthroplasty.

## 9. Biologic Resurfacing Options

Resurfacing arthroplasty using interpositional graft offers potential advantages over conventional TSA, including the ability to biologically resurface the glenoid, in addition to biologic or nonbiologic resurfacing of the humeral head, thereby avoiding complications of TSA in cases of bipolar disease.

Several options are available for glenoid resurfacing. Interpositional grafts may be secured over the glenoid, thereby offering a biologic surface that articulates with the humeral head. The use of a lateral meniscus as a biologic interpositional graft in the glenoid has been described using both open and arthroscopic techniques [36–38]. Studies have shown that the lateral meniscus provides better glenohumeral coverage with reduced peak forces and contact stress compared to the medial meniscus in the shoulder [39]. Other examples of biological interpositional resurfacing of the glenoid include Achillestendon allograft, autogenous fascia lata, anterior shoulder capsule, the Restore patch (DePuy Orthopaedics, Warsaw, IN, USA), and the GraftJacket (donated human skin; Wright Medical Technology, Arlington, TN, USA).

 Krishnan and colleagues published a prospective study analyzing biologic resurfacing of the glenoid using a variety of interpositional tissues for grafts, including Achillestendon allograft (18 shoulders), anterior capsule (7 shoulders), and autogenous fascia lata (11 shoulders) [40]. Their early results were comparable to results of TSA without the inherent risks of arthroplasty, with significant increases in mean ASES scores in 31 of 36 patients and no revisions for humeral component loosening. They suggested that Achillestendon allograft is the preferred graft type for biologic resurfacing. In a study of six patients, Burkhead and Hutton performed porous-coated humeral head hemiarthroplasty along with glenoid resurfacing using autogenous fascia lata or anterior shoulder capsule and found good or excellent results in all patients after two years [36]. Others have reported good results after arthroscopic glenoid resurfacing using the Restore patch, an implant made of porcine small intestine submucosal cells with potentially pluripotent properties, with the hope of regenerating viable chondrocytes and a matrix of hyaline cartilage on the surface of the glenoid [41]. Finally, Bhatia described an arthroscopic procedure for resurfacing the glenoid with the GraftJacket, a regenerative tissue matrix consisting of processed 1 to 2 mm thick human donor skin that retains native proteins, collagen, and vascular channels [37].

However, not all authors have reported favorable results following hemiarthroplasty combined with biologic interposition, particularly at longer-term followup. Nicholson et al. recently published a series of thirty young, high-demand patients with bipolar defects who were treated with a biologic interpositional lateral meniscus glenoid allograft and uncemented hemiarthroplasty [42]. Their short-term results overall were good, with all patients demonstrating significant improvement in outcome scores after 18 months. However, they did report a 17% complication rate within the first year, all requiring reoperation, as well as two patients with graft failure requiring conversion to a polyethylene glenoid component [42]. Others have also shown comparable findings. Elhassan and colleagues performed a review of 13 patients with an average age of 34 years and average followup of 48 months undergoing soft-tissue resurfacing of the glenoid with a concomitant humeral head arthroplasty. The authors found that ten of the thirteen patients required a revision total shoulder arthroplasty at a mean of 14 months postoperatively and concluded that the procedure has poor outcomes in patients under 50 years [43]. Verma et al. also noted a high clinical failure rate (51.2%) in 45 patients undergoing biologic resurfacing of the glenoid with lateral meniscus allograft or human acellular dermal tissue matrix [44]. An unacceptably high failure ultimately led the authors to recommend against the procedure's utility in young, active patients with glenohumeral arthritis.

These aforementioned techniques are currently investigational in nature and may serve as a temporal bridge for young or middle-aged active patients who are not yet candidates for TSA. Long-term studies demonstrating success and failure rates are currently lacking, as are randomized, controlled trials comparing biologic resurfacing techniques with hemiarthroplasty, TSA, or other palliative restorative procedures.

## 10. Postoperative Rehabilitation

The postoperative regimen is important to the success of the cartilage repair process. With all procedures, early range of motion is encouraged to increase circulation and promote healing, but with some limits to protect the repair site. Patients who have undergone palliative treatment strategies should begin physical therapy immediately, with range of motion and strength exercises restricted only by pain. If a capsular release has also been performed, patients should receive physical therapy 3 times per week for 6 to 8 weeks.

Patients who had reparative or restorative procedures should perform 600 cycles of pendulum exercises daily for the first 6 weeks to stimulate the healing response [9]. Active range of motion exercises can be started at 6 weeks. However, animal studies have found that fibrocartilage may not reach a significant degree of maturity until 12 weeks postoperatively, which suggests that longer periods of protected range of motion may be beneficial [45].

For reconstructive treatment or any procedures which require takedown and repair of the subscapularis, a rehabilitation protocol similar to that for arthroplasty should be followed. No active internal rotation or extension is allowed for the first 6 weeks post-operatively. Passive-to-active range of motion is gradually advanced to prespecified limits for external rotation (40 degrees), flexion (120 degrees), and abduction (75 degrees). At 8–12 weeks, range of motion can be increased as tolerated and strengthening exercises are started. Patients typically progress to full, unrestricted activities by 6 months, although some cases may take longer to observe the full benefit.

## 11. Conclusion

Treatment of glenohumeral cartilage injuries in young patients continues to be a challenging issue. Surgeons must differentiate incidental chondral lesions from symptomatic lesions that are responsible for the patients' pain. Even with appropriate imaging and a thorough patient history and physical examination, this remains difficult and often results in a diagnosis of exclusion that can only be verified on arthroscopy. After identification of a symptomatic patient, treatment depends on several factors, including the patient's age, comorbidities, degree of injury, concomitant shoulder pathology, expectations, and activity level. Patients with lower functional demands may experience success with nonoperative measures such as medication and injections or arthroscopic surgery involving debridement and capsular release. Young patients with higher functional demands or extensive arthritis may require more aggressive surgical treatments that restore or reconstruct the cartilage. A variety of innovative nonarthroplasty procedures have been utilized for glenohumeral arthritis in the young patient, with promising short- to midterm success. However, long term outcomes or randomized trials are lacking, and future work is required to determine appropriate indications for each procedure and predictors for lasting success.

## Figures and Tables

**Table 1:** Outcomes of palliative and reparative treatment for glenohumeral arthritis.

Author	Surgical technique	Number of patients	Main results	Other notable findings
Van Thiel et al. [13]	Palliative	71	Significant improvement in pain, SST score, and range of motion in short term	22% went on to shoulder replacement in 10.1 months
Cameron et al. [22]	Palliative	61	Significant improvement in pain at 28 months	Workers' Compensation patients fared poorly
Weinstein et al. [26]	Palliative	25	84% had good or excellent findings at 30 months	Poor results associated with severe joint incongruity or large osteophytes
Millett et al. [25]	Reparative	25	Significant improvement in pain, ASES score, ability to work	Best results in those with isolated humeral lesions
Frank et al. [18]	Reparative	16	Significant improvements in pain, ASES score, and SST score at 27.8 months	3 patients had failed results
Snow and Funk [27]	Reparative	6	Significant improvement in constant score	Repeat arthroscopy confirmed good filling of lesions with fibrocartilage
